# Social information affects Canada goose alert and escape responses to vehicle approach: implications for animal–vehicle collisions

**DOI:** 10.7717/peerj.8164

**Published:** 2019-12-17

**Authors:** Bradley F. Blackwell, Thomas W. Seamans, Travis L. DeVault, Steven L. Lima, Morgan B. Pfeiffer, Esteban Fernández-Juricic

**Affiliations:** 1 National Widlife Research Center, Wildlife Services, U.S. Department of Agriculture, Sandusky, OH, USA; 2 Department of Biology, Indiana State University, Terre Haute, IN, USA; 3 Department of Biological Sciences, Purdue University, West Lafayette, IN, USA

**Keywords:** Animal–aircraft collisions, Animal–vehicle collisions, Escape behavior, Flight-initiation distance, Perceived risk, Social information

## Abstract

**Background:**

Animal–vehicle collisions represent substantial sources of mortality for a variety of taxa and can pose hazards to property and human health. But there is comparatively little information available on escape responses by free-ranging animals to vehicle approach versus predators/humans.

**Methods:**

We examined responses (alert distance and flight-initiation distance) of focal Canada geese (*Branta canadensis maxima*) to vehicle approach (15.6 m·s^−1^) in a semi-natural setting and given full opportunity to escape. We manipulated the direction of the vehicle approach (direct versus tangential) and availability of social information about the vehicle approach (companion group visually exposed or not to the vehicle).

**Results:**

We found that both categorical factors interacted to affect alert and escape behaviors. Focal geese used mostly personal information to become alert to the vehicle under high risk scenarios (direct approach), but they combined personal and social information to become alert in low risk scenarios (tangential approach). Additionally, when social information was not available from the companion group, focal birds escaped at greater distances under direct compared to tangential approaches. However, when the companion group could see the vehicle approaching, focal birds escaped at similar distances irrespective of vehicle direction. Finally, geese showed a greater tendency to take flight when the vehicle approached directly, as opposed to a side step or walking away from the vehicle.

**Conclusions:**

We suggest that the perception of risk to vehicle approach (likely versus unlikely collision) is weighted by the availability of social information in the group; a phenomenon not described before in the context of animal–vehicle interactions. Notably, when social information is available, the effects of heightened risk associated with a direct approach might be reduced, leading to the animal delaying the escape, which could ultimately increase the chances of a collision. Also, information on a priori escape distances required for surviving a vehicle approach (based on species behavior and vehicle approach speeds) can inform planning, such as location of designated cover or safe areas. Future studies should assess how information from vehicle approach flows within a flock, including aspects of vehicle speed and size, metrics that affect escape decision-making.

## Introduction

Animal–vehicle collisions (involving ground and air traffic) are sources of mortality for a variety of taxa and can even pose hazards to humans and their property (Conover, 2002; Bishop & Brogan, 2013; Lima et al., 2015; Hill, DeVault & Belant, 2019). Animals have been shown to engage in escape behavior when approached by vehicles (e.g., aircraft; Bernhardt et al., 2010), which has prompted the use of antipredator behavior theory to frame empirical research (DeVault, Blackwell & Belant, 2013; Lima et al., 2015). Yet, animal escape behavior towards non-predatory threats has been traditionally studied via human observers walking towards animals (Ydenberg & Dill, 1986; Fernández-Juricic et al., 2005; Li et al., 2011), with flight-initiation distance (FID, distance between prey and approaching threat at which point the prey initiates escape) serving as the metric of perceived risk (Ydenberg & Dill, 1986; Cooper & Frederick, 2007). However, vehicles move at much faster speeds than predators or humans, which can influence the animal’s initiation of the escape response in ways not predicted by antipredator behavior theory (DeVault et al., 2015).

The vast differences in the speed of a human/predator versus vehicle approach creates a challenge in the empirical study of animal–vehicle collisions (Blackwell et al., 2019). From a logistical point of view, mimicking a realistic high-speed vehicle approach to assess the animal response is constrained by the fact that approach vehicles must deviate to avoid collisions with the animals (Blackwell et al., 2009). This problem has been solved by using a virtual simulator in which birds in a small enclosure are exposed to videos of a vehicle approaching at different speeds (DeVault et al., 2015, 2017, 2018a), in much the same way as done for human studies on street crossing (Dommes & Cavallo, 2011). These experiments, however, are constrained by the fact that animals are confined within relatively small test enclosures, having no opportunity to display full escape behavior after initiating flight in response to the stimulus (Sheridan et al., 2015).

We suggest, therefore, that in the context of experiments involving captive birds exposed to vehicle approach the opportunity to “escape” by becoming airborne can influence our ability to discern not only treatment effects on alert and escape behavior, but also the carryover of perceived risk into post-escape responses. This is important because animals have been found to modulate post-escape responses to the level of risk perceived during the approaching threat situation (Fernández-Juricic et al., 2006). In other words, the metric of perceived risk, FID, serves as only a proxy to understanding how animals escape. Ultimately, a better understanding of how species detect and respond to vehicle approach can lay the foundation for methods to mitigate animal–vehicle collisions (Lima et al., 2015; Blackwell et al., 2016).

The purpose of our study was to quantify the responses of flight-capable Canada geese (*Branta canadensis maxima*), a highly social species, to vehicle approach when given full opportunity to escape. Our specific objectives were to quantify alert distance, a proxy of detection relative to an approaching threat (Fernández-Juricic, Jimenez & Lucas, 2001), FID and type of escape in response to vehicle approach direction and the availability of social information about the approach in the flock. We manipulated the direction (direct or tangential) of the vehicle approaching a single individual (focal bird) and whether or not two geese composing a companion group had visual information about the vehicle approaching. These two factors have not been tested before simultaneously despite their relevance in daily vehicle–wildlife interactions. For instance, animals often use space close to roads or runways relative to the location of resources, despite approaching vehicles (Lima et al., 2015; Blackwell et al., 2019), leading to either direct or tangential interactions with vehicles. Further, in social species some individuals in the flock (i.e., detectors) might first detect the vehicle and their response can influence escape behavior of the rest of the flock (i.e., non-detectors), as has been shown in predator-prey contexts (Lima, 1995a, 1995b; Lima & Zollner, 1996; Fernández-Juricic & Kowalski, 2011).

We hypothesized that focal birds would perceive greater risk (i.e., show greater FID) from a directly approaching vehicle versus a tangential approach (Burger & Gochfeld, 1981, 1990; Frid & Dill, 2002; Stankowich & Blumstein, 2005; DeVault et al., 2017). Specifically, we predicted that birds would show no difference in alert response based on approach direction, but escape at greater distances and be more likely to take flight from the experimental arena when the vehicle approached directly rather than tangentially. We also hypothesized that if all three birds (focal + companion group) could visually detect the approaching vehicle, information about the threat would become available to the focal bird sooner than if only the focal bird (but not the companion group) was able to see the vehicle (Lima & Zollner, 1996). As such, we predicted that the focal bird would become alert and escape at greater distances, as well as be more likely to take flight and leave the experimental arena when it had access to social information about the approaching vehicle via the companion group, as compared to when it had to rely solely on personal information (i.e., companion group not having visual access to the vehicle approach).

## Materials and Methods

### Test species

We used the Canada goose as our test species. Although records of Canada goose collisions with ground-based vehicles are few (e.g., representing <0.10% of specimens reported as recovered in North America through 2010; Bishop & Brogan, 2013; and not represented in more recent statistics; Loss, Will & Marra, 2014), the species has been involved in approximately 1.5% of all known-species bird–aircraft collisions (strikes) reported to the FAA (1990–2018; Dolbeer et al., 2019). Notably, >38% of bird strikes to civil aircraft in the US or aircraft registered to the US occurred at zero feet above ground level (Dolbeer et al., 2019). Given its body mass (on average 3.6 kg) and likelihood of causing damage to the aircraft (48–50% of strikes; DeVault et al., 2018b; Dolbeer et al., 2019), the species ranks second among birds in strike risk across the US in a recent estimate involving civilian and commercial aircraft (DeVault et al., 2018b). In the context of strike hazard (i.e., associated cost and damage when struck), the Canada goose ranks third in strikes involving U.S. military aircraft (Pfeiffer, Blackwell & DeVault, 2018).

In late June 2017 we received 121 giant (urban) Canada geese from the Ohio Department of Natural Resources, Division of Wildlife, captured as “nuisance” geese during summer molt phase. As urban geese, these birds presumably had been frequently exposed to vehicle traffic. We held the geese outside in a natural area (10 m × 43 m) protected on the sides and overhead (approximately 2.7 m high) by netting. The birds had access to grass, shade, and a 9 m × 5 m, fenced area of a natural pond. We supplemented natural forage with poultry pellets and whole kernel corn ad libitum.

### Study area

**Figure 1:**
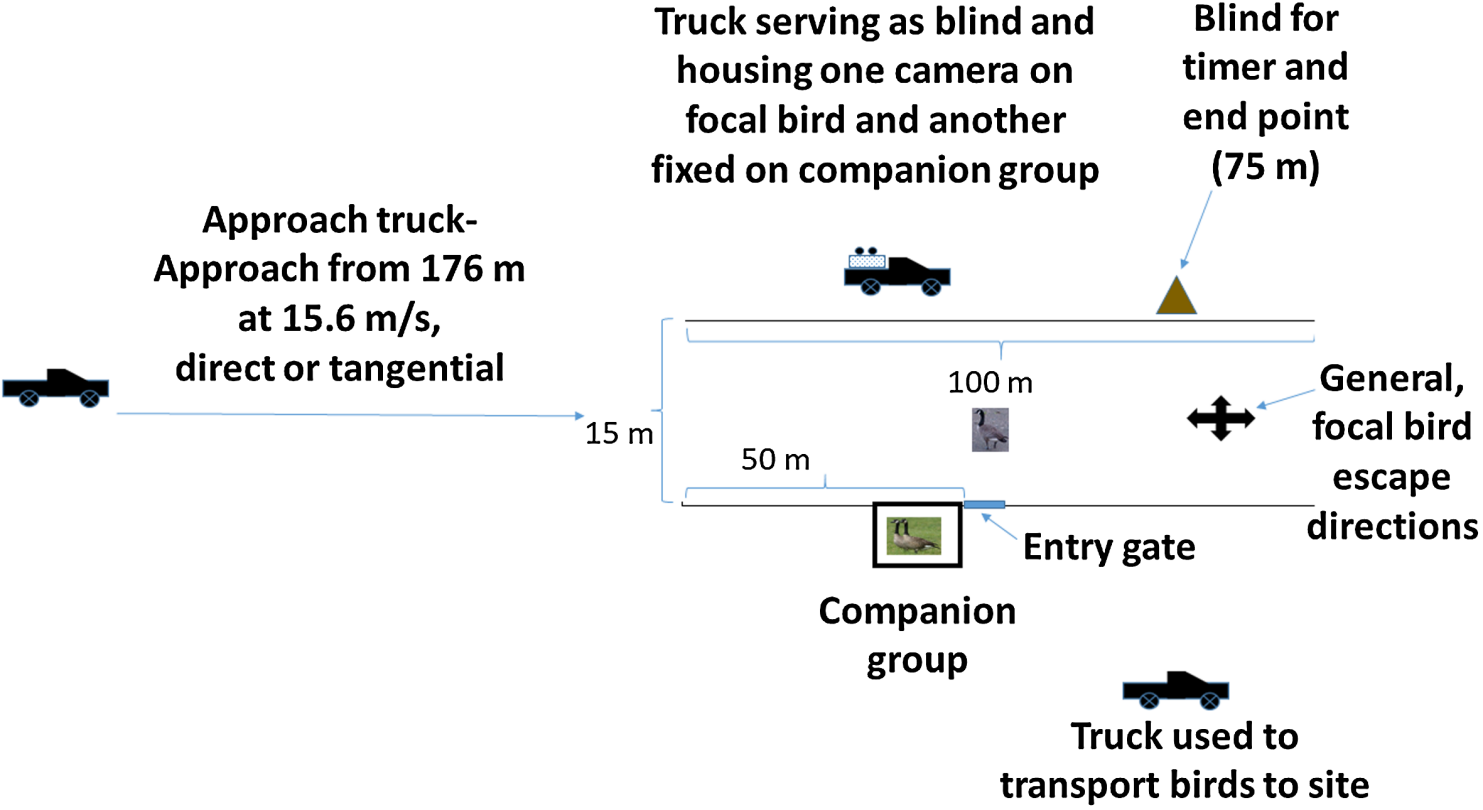
Schematic representation of the experimental arena. An individual, flight-capable Canada goose (focal bird) and two birds composing a companion group and held in an enclosure, were exposed to vehicle approach in an experiment conducted 1–4 August 2017 at the NASA Plum Brook Facility in Erie County, OH, USA. Photographs of geese were taken by USDA employes.

We conducted our study on the 2,200 ha National Aeronautics and Space Administration Plum Brook Station (PBS), Erie County, OH, USA (41°22′N, 82°41′W) during August 2017. The PBS comprises a mix of old field, grasslands, open woodlands, mixed hardwood forest and anthropogenic structures segmented by numerous access roads (see land cover description by Bowles & Arrighi, 2004; DeVault et al., 2014). Our experimental site comprised a 100 m long grass chute, bordered on each side by a 1.5 m high, orange plastic snow fence. The chute was 15 m wide, open at both ends during treatment and lacking a top, and allowed freedom of movement by the geese and passage of a full-size pickup truck (Fig. 1). Specifically, a goose fleeing from the vehicle approach potentially had ≥90° extending from each side and ahead of the vehicle approach path (i.e., a 180° semicircle) to select an escape direction. We also considered the possibility that some birds might escape at angles opposite the vehicle approach path.

We included a 2.4 m × 2.4 m × 1.8 m cage adjacent to, but outside of the chute and positioned at the 50 m point of the chute (Fig. 1); this cage held two birds composing the companion group. We included the companion group in our design because the Canada goose is highly social (Raveling, 1969) and to provide a calming effect and possible source of social information for focal birds released into a novel experimental setting (Devereux et al., 2005). To facilitate blind versus exposed approaches for the companion group, we fit a removable, opaque fabric screen across the side of the cage facing the vehicle. The screen was in place for blind approaches and removed for exposed approaches.

Next to the companion group cage, at the 50 m point, we installed a moveable flap in the fencing (Fig. 1); here, we positioned a carrying cage and allowed each focal bird to enter the chute on its own volition. Opposite the companion group and on the far side of the chute, we positioned one of three pickup trucks used in the experiment. Here, a truck was fitted with a fabric screen along the frame of the truck bed on the chute side; an observer operated two digital video cameras from this point, one fixed on the companion group and the other following the focal bird. At the 75 m point and opposite the side with the companion group, we positioned another screen from which an observer could note the time when the approach vehicle reached 75 m, the endpoint of an approach. We positioned the approach vehicle (white Ford F-150 pickup truck) 176 m from the chute entrance (Fig. 1). To aid in estimating bird position at the point of alert response, we marked five m intervals along the fence using black tape.

### Treatments

Our treatments included direct and tangential approaches against 80, single, focal birds (*N* = 80 approaches in a factorial design, with 20 approaches per approach/companion group exposure level (direct approach/companion group blind to the vehicle approach, direct approach/companion group exposed to the vehicle approach, tangential approach/companion group blind, tangential approach/companion group exposed). The companion group was present in the holding cage (Fig. 1) for 10 consecutive approaches (five approaches/exposure level; Table 1). We randomly selected the first treatment (approach and exposure level), but incorporated a systematic approach thereafter to provide a level of efficiency relative to necessary changes to the holding cage for exposure level and to save potential confusion in communication between observers and the driver. Inherently, this systematic approach resulted in the introduction of a new, naïve companion group at the same exposure (i.e., blind in our experiment) through run 60; however, we considered the effects of companion group identity in our analyses (see below). After the random selection of the first treatment combination, we repeated that treatment combination for another four approaches. We then systematically moved to the next exposure level (blind or exposed) for the same companion group, but maintained the directionality of the approach (Table 1).

**Table 1:** Treatment scenario for replicates. Treatment scenario for replicates (order shown for replicates 1–20, but applicable through replicate 60) involving vehicle approach against single, focal birds and a companion group. A companion group (2 birds) was exposed to 10, repeated vehicle approaches (*n *= 5 blind and *n* = 5 exposed). The experiment was conducted in Erie County, OH, USA from 1 to 4 August 2017. See text for more detail on the experimental design and protocol.

Treatment	Approaches 1–5	Approaches 6–10	Approaches 11–15	Approaches 16–20
Direct/companion group blind	5 Naïve focal birds exposed individually	–	–	–
Direct/companion group exposed	–	5 Naïve focal birds exposed individually	–	–
Tangential/companion group blind	–	–	5 Naïve focal birds exposed individually	–
Tangential/companion group exposed	–	–	–	5 Naïve focal birds exposed individually

After the 10th approach for a companion group, the birds were replaced with two birds naïve to the experimental setting and we moved to the next approach scenario. This sequence of approaches began with the approach scenario not selected in our first series of five approaches (Table 1). Again, we conducted five approaches for this treatment combination before changing the exposure level of the companion group. After the next five approaches (replicate 20; Table 1), we once again replaced the companion group with two naïve birds. We maintained this systematic selection of treatments through replicate 60. However, for replicates 61 through 80 we shifted our treatment order because of threatening weather. We conducted 10 approaches, five as tangential/companion group exposed (the treatment that we would have followed had we adhered to the original systematic approach) and another five as direct/companion group exposed. We then conducted a final 10 approaches, five as tangential/companion group blind and five as direct/companion group blind. Ultimately, we used 16 birds in eight companion groups.

### Protocol

We conducted our experiment between 07:40 h and 11:00 h, 1–4 August 2017. All birds used in our experiment were captured via hand net from the holding area and transferred approximately 90 m to the experimental site in single-animal carriers via vehicle. The two birds composing the companion group were captured and transferred to the holding cage (Fig. 1) immediately preceding the introduction of the first focal bird and initiation of a sequence of 10 approaches. For each focal bird, the handler positioned the carrier in the cage opening (Fig. 1), opened the door to the carrier and then returned to the vehicle (pickup truck). The bird was allowed to enter the chute on its own. In instances where birds refused to leave the carrier after 2 min, the handler gently flushed the bird from the carrier, then returned to the vehicle.

Because a focal bird had recently been handled, possibly flushed from the carrier and was in a novel situation, the animal was at an elevated alert status, but able to escape. Therefore, we did not allow for an acclimation period. Instead, when the bird exited the carrier and began exploring the chute area in a normal manner (i.e., without showing erratic or panic behavior), the camera operator (Fig. 1) contacted the timer via radio to prepare the clock and alert the driver. The timer then contacted the driver via radio, began a three-step countdown and initiated a digital stopwatch at the start. The audio from the timer was also recorded on the two cameras, as was a visual indicator at the camera lens that the approach had begun. We used two Sony High Definition Handycam video cameras with a resolution of 1,080 × 1,920 pixels and a recording speed of 30 frames·s^−1^ in high-definition, MPEG Transport Stream format. One camera was fixed on the companion group, while we followed the focal bird with the other. We recorded each approach to document bird behavior in relation to distance and time from the approaching vehicle.

At the timer’s command, the driver quickly accelerated from 0 m·s^−1^ to 15.6 m·s^−1^ and directed the vehicle either at a bird (direct approach) or tangentially (within three m of the focal bird). The driver held the original approach direction, even when the focal bird changed its position. In other words, we did not chase the bird, but maintained our original course. An approach was complete after the front of the truck reached the timer’s position 75 m from the chute entrance (Fig. 1). In instances where a bird failed to move or stepped in front of the vehicle, the driver maintained speed and direction as long as possible to allow for an escape response. When visibility with the bird was lost, the driver braked or swerved to attempt to avoid a collision.

Because we recorded approach time for each replicate, we were able to calculate average speed for each approach. Also, at our approach speed (≤15.6 m·s^−1^) we did not anticipate striking a focal bird. However, by introducing a freely moving, flight-capable bird into the experiment so as to achieve realism in how geese interpret and respond to vehicle approach, there was an inherent risk of a collision. Therefore, in the event of a collision, an observer, who could reach an injured bird within 30 s, was present with equipment approved for euthanizing a Canada goose (via blunt force trauma to the head using a Brock Industries TED Captive Bolt Dispatch Tool, http://www.bock-industries.com/our-company.html; required in only one instance). Our animal care methods and experimental protocols were reviewed and approved by the National Wildlife Research Center’s Institutional and Care and Use Committee via QA-2795 and in consideration of Fair, Paul & Jones (2010) and the American Veterinary Medical Association (2013).

### Behavioral metrics

Research pertaining to vehicle approach and likelihood of animal–vehicle collision has consistently used units of time-to-collision to express alert and flight responses (Blackwell et al., 2009, 2012; Blackwell, Seamans & DeVault, 2014; Doppler et al., 2015; DeVault et al., 2014, 2015), particularly when assessing margins of safety (e.g., spatial, temporal, or dynamic; DeVault et al., 2015). Time-to-collision metrics are more meaningful, however, when combined with an estimate of animal escape time (time necessary for successful escape) relative to an escape behavior (e.g., flight) over an a priori escape distance and the speed of the approaching vehicle (DeVault et al., 2015). In this experiment, we did not define a particular escape behavior, because geese might step, run, or fly at various angles to avoid vehicle approach. Further, we did not decide a priori on an escape distance relative to a particular behavior and vehicle approach speed. Instead, we based our analyses on alert distance and FID. Also, because geese will use low, guttural vocalization prior to flight, with a transition to loud honking at takeoff (Raveling, 1969), aural communication in escape decision-making during our experiment was likely. Given the role of vocalizations in goose communications, the fact that we noted no alarm calls (Whitford, 1998) and that we could not discern lower frequency calls, we did not attempt to control for aural communication between the focal bird and companion group.

We used Handbrake, an open source video transcoder, to convert our videos and audio to M4V format. We then analyzed our videos using Adobe Premier Pro CC 2018. Again, we note each focal bird was at an elevated stage of alert upon entry into the chute. Therefore, we considered an alert response to vehicle approach as behavior that transitioned to a heightened alert level, generally indicated by the bird extending its neck fully, slowing or ceasing movement, and turning its head to allow a monocular view, thus placing a greater area of high retinal acuity on the approaching vehicle (Fernández-Juricic et al., 2011). We defined flight response as behavior that involved an obvious intent to avoid the approaching vehicle, whether by stepping to the side, walking or running from the vehicle, or becoming airborne. We recorded FID, but also characterized subsequent escape behavior simply as steps/run, actual flight, or no response.

Based on the camera clock (i.e., min:sec:frames) and frame rate, as well as the recorded approach and start time, we estimated the time into approach of alert and flight responses for the focal bird to 0.01 s. We used the five m interval markers on the chute fence to provide us with an approximate straight-line distance of a focal bird from the 75 m point, the end of an approach. We then used the time into approach at alert and average vehicle speed (calculated as 251 m/total approach time) to calculate distance traveled by the vehicle at the point of alert (see Fig. 1). We subtracted the position of the focal bird relative to the 75 m point of the chute (i.e., the end point of the approach) from the position of the vehicle relative to the chute end point to obtain the alert distance, or the distance between the focal bird and vehicle at the point of alert. We followed the same steps to obtain FID. When birds failed to show alert behaviors, but initiated escape response, we scored alert distance = FID. If birds failed to show alert and failed to initiate escape, both metrics were scored as zero (Blackwell et al., 2009, 2012). For both metrics, greater values indicate an earlier response to the approaching vehicle, hence greater perceived risk.

In instances where the driver braked or swerved to avoid a collision, we could not always locate the truck in a video frame prior to the driver taking the evasive action. As such, we could not accurately estimate truck position prior to the evasive action and, thus, the average approach speed. We also recognized that average approach speed would be lower in the event of an evasive maneuver. Therefore, we opted to calculate the average approach time across complete approaches for the given day and use this value in calculating approach speed (i.e., 251 m approach distance/approach time) for the replicate that involved the driver’s evasive action.

After each approach we recorded a 15 s ambient light intensity (μmol m^−2^ s^−1^) at the location where the focal bird showed alert response, using a Li-C or LI-250 Light Meter and LI-190SA Quantum Sensor (Lincoln, NE, USA). Sunlit patches have been shown to limit prey detection of predators (disability glare hypothesis; Fernández-Juricic et al., 2012). Because our approaches were always tangential to the companion group and half of these involved birds blind to the approach, we did not measure a separate light intensity at the companion group cage. We also recorded wind speed and air temperature with a Kestrel 4500 Pocket Weather Tracker (Nielson-Kellerman, Boothwyn, PA, USA). Raw data are provided in Data R1.

### Statistical analysis

We first examined whether focal bird alert distance and FID responses differed with regard to whether the focal bird was flushed from the holding cage or entered the chute voluntarily. We used a linear mixed model (via PROC MIXED, SAS 9.2; SAS, Inc., Cary, NC, USA), Residual Maximum Likelihood as the estimation method, a random intercept with subject = companion group identity (ID) and a categorical variable representing whether the bird was flushed or not as the fixed effect. The random intercept served to assess the effect on focal bird responses due to a specific companion group, as well as the exposure of that group to 10 repeated approaches. We used Kenward-Roger degrees of freedom and assigned the covariance structure as autoregressive. We also examined the distribution of model residuals relative to an assumption of normality (Code S1).

An important consideration in our use of adjusted approach times (see above) in calculating approach speed was to understand the possible differential effects on unadjusted versus adjusted approach times of the following variables and interaction: approach direction, whether or not the companion group was exposed visually to the approaching vehicle, the interaction of approach direction and companion group exposure level, and companion group ID. Therefore, we examined the fixed effects of approach direction, companion group exposure level and the interaction between these two categorical factors on unadjusted and adjusted approach times. For both analyses, we again used a linear mixed model, Residual Maximum Likelihood as the estimation method and a random intercept with subject = companion group ID, Kenward-Roger degrees of freedom, and the covariance structure as autoregressive (Code S1). We also included ambient light intensity as a fixed effect in the model as it has been previously found to affect alert and escape responses to predators (Fernández-Juricic & Tran, 2007; Fernández-Juricic et al., 2012). Wind speed (Pearson product moment correction, *r* = −0.27, *P* = 0.014) and ambient temperature (Pearson product moment correction, *r* = 0.752, *P* < 0.001) were correlated with ambient light intensity, therefore we removed them from this and all subsequent models to avoid collinearity issues. Again, we examined the distribution of model residuals relative to an assumption of normality (Code S1).

For focal-bird alert and flight responses, we also assessed the effects of approach direction, companion group exposure level, the interaction between these later factors and ambient light intensity by using a linear mixed model (Code S1). As before, we selected Residual Maximum Likelihood as the estimation method, a random intercept with subject = companion group ID, Kenward-Roger degrees of freedom and the covariance structure as autoregressive. We also examined the distribution of model residuals relative to an assumption of normality (Code S1).

Finally, we assessed the effects of vehicle approach direction and whether the companion group was exposed visually or not to the approaching vehicle (and their interaction) on the type of focal bird escape behaviors (step/run or flight) using a generalized linear mixed model (PROC GLIMMIX, SAS; Code S1). We categorized the response factor in the following scale: 0 (no escape), 1 (step/run) and 2 (flight). PROC GLIMMIX models the probability of the lower end of this scale (e.g., no escape in this case). We followed Kiernan (2018) to interpret the results of this multinomial model. As before, we also added ambient light intensity as a potential confounding factor. We used a multinomial distribution, a logit link function and a random intercept with subject = companion group ID. For all models, we evaluated variable contribution to variance of the response variable based on α = 0.05.

## Results

We conducted 88 vehicle approaches involving 88 focal birds and 16 birds composing eight companion groups (*N* = 104 birds). However, we did not consider eight focal birds, as they escaped from the experimental site immediately after release into the chute, prior to the initiation of the vehicle approach. The 80 completed approaches comprised 20 approaches per approach direction and companion group exposure level. During these completed approaches, it was necessary to flush the focal bird from the holding cage on 14 occasions (direct approach/companion group mates blind to approach = four instances; direct/companion group exposed to approach = two instances; tangential/companion group blind to approach = five instances; tangential/companion group exposed to approach = three instances). We found no difference in alert or FID between birds that were not flushed into the chute and those that were flushed (Table 2). Therefore, we pooled the data for the analyses.

**Table 2:** Results from a linear mixed model analysis. Results from a linear mixed model analysis of (A) alert distance and (B) flight-initiation distance (FID) shown by flight-capable Canada geese exposed to vehicle approach, 1 through 4 August 2017, at the NASA Plum Brook Facility in Erie County, OH, USA. Treatments comprised vehicle approach direction (direct or tangential) and exposure level of the companion group (blind or exposed; CGtreat) to the approaching vehicle. Here, we also considered the effect of flushing a bird from a holding cage on escape response.

Effect	Num df	Den df	Chi-Square	*P* > Chi-square	*F*	*P > F*
(A) Alert distance						
Flushed	1	73.4	0.03	0.8706	0.03	0.8710
Direction	1	18.1	0.08	0.7766	0.08	0.7799
CGtreat	1	47.9	0.56	0.4533	0.56	0.4569
Direction × CGtreat	1	74.8	0.09	0.7654	0.09	0.7663
(B) Flight-initiation distance						
Flushed	1	74.8	0.00	0.9765	0.00	0.9766
Direction	1	28.5	1.02	0.3114	1.02	0.3199
CGtreat	1	62.5	0.09	0.7594	0.09	0.7604
Direction × CGtreat	1	74.2	0.52	0.4703	0.52	0.4725

The driver took some evasive action (braking or swerving) on nine occasions. In review of the videos, however, we could discern evasive maneuvers that might have affected approach speed in only four instances (direct approach/ companion group exposed = three instances; direct approach/companion group blind = one instance). Considering all approaches, and without adjusting times for an evasive action, mean approach time (over the 251 m) varied little relative to that for complete approaches (all approaches = 19.8 s, SD = 0.7 s; complete approaches = 19.7 s; SD = 0.5 s). For the four instances when the driver took evasive actions, approach times increased by 0.44–2.42 s ($\bar{X}$ increase = 1.6 s, SD = 0.9 s; $\bar{X}$ adjusted time = 19.6 s, SD = 0.1 s) beyond the average approach time for complete approaches on a respective day. When we adjusted for evasive actions (as described above), mean approach time across all treatment combinations was 19.7 s (SD = 0.6 s).

Our models for unadjusted and adjusted approach times failed to converge when including ambient light. Therefore, we removed this variable from the analyses (Code S1). In addition, model residuals were not normally distributed, despite attempts to transform the response variable. However, linear and linear mixed effects models are quite robust to violation of assumptions regarding normality of model residuals (Knief & Forstmeier, 2018 and citations therein). Specifically, Knief & Forstmeier (2018) noted that *P*-values from Gaussian models are quite robust to even substantial violation of the assumption of normality, but that bias generally occurs if the dependent variable or predictors are heavily skewed, resulting in outliers. In our study, the mean unadjusted and adjusted approach times fell within 0.1 s of each other and variation was low within each response variable (see below).

We found that neither unadjusted approach times nor our data when considering the four instances of adjusted approach times varied relative to approach direction (unadjusted times, direct approach: $\bar{X}$ = 19.8 s, SD = 0.8 s; tangential: $\bar{X}$ = 19.7, SD = 0.5 s; *F*_1,76_ = 1.45, *P* = 0.232). We found similar results for our adjusted approach times (direct approach: 19.7 s, SD = 0.6 s; tangential: 19.7 s, SD = 0.5 s; *F*_1, 76_ = 0.01, *P* = 0.913). Further, we found no effect of whether or not the companion group was exposed to the vehicle approach, though the effect on the unadjusted time was marginally nonsiginficant (unadjusted times blind: $\bar{X}$ = 19.6, SD = 0.5 s; exposed: $\bar{X}$ = 19.9 s, SD = 0.7; *F*_1,76_ = 3.59, *P* = 0.062; adjusted times, blind: $\bar{X}$ = 19.6 s, SD = 0.5 s; exposed: $\bar{X}$ = 19.7 s, SD = 0.5 s; *F*_1,76_ = 1.26, *P* = 0.266). Here, we note that the three instances of evasive action by the driver that occurred during direct approaches with the companion group exposed increased the approach time on average by 2.01 s (SD = 0.51 s). In contrast, the single instance of evasive action for a direct approach with companion group blind resulted in a 0.44 s increase in approach time. Also, we observed no interaction effect between approach direction and companion group exposure level for either unadjusted (*F*_1,76_ = 0.74, *P* = 0.392) or adjusted approach times (*F*_1,76_ = 0.01, *P* = 0.903). For both analyses, the Null Model Likelihood Ratio test indicated no effect of the random intercept for companion group ID (*χ*^2^ = 0.00, *P* = 1.000).

Despite the driver taking evasive actions to avoid striking focal geese (but without compromising safety), the vehicle unfortunately physically contacted five focal birds (e.g., some involving a brush of a wing as the bird took flight) during only direct approaches. Four of these five birds nevertheless escaped the experiment site by flying soon after the vehicle passed or in response to a capture attempt by the observer; the fifth (clearly injured) bird was euthanized immediately.

**Figure 2:**
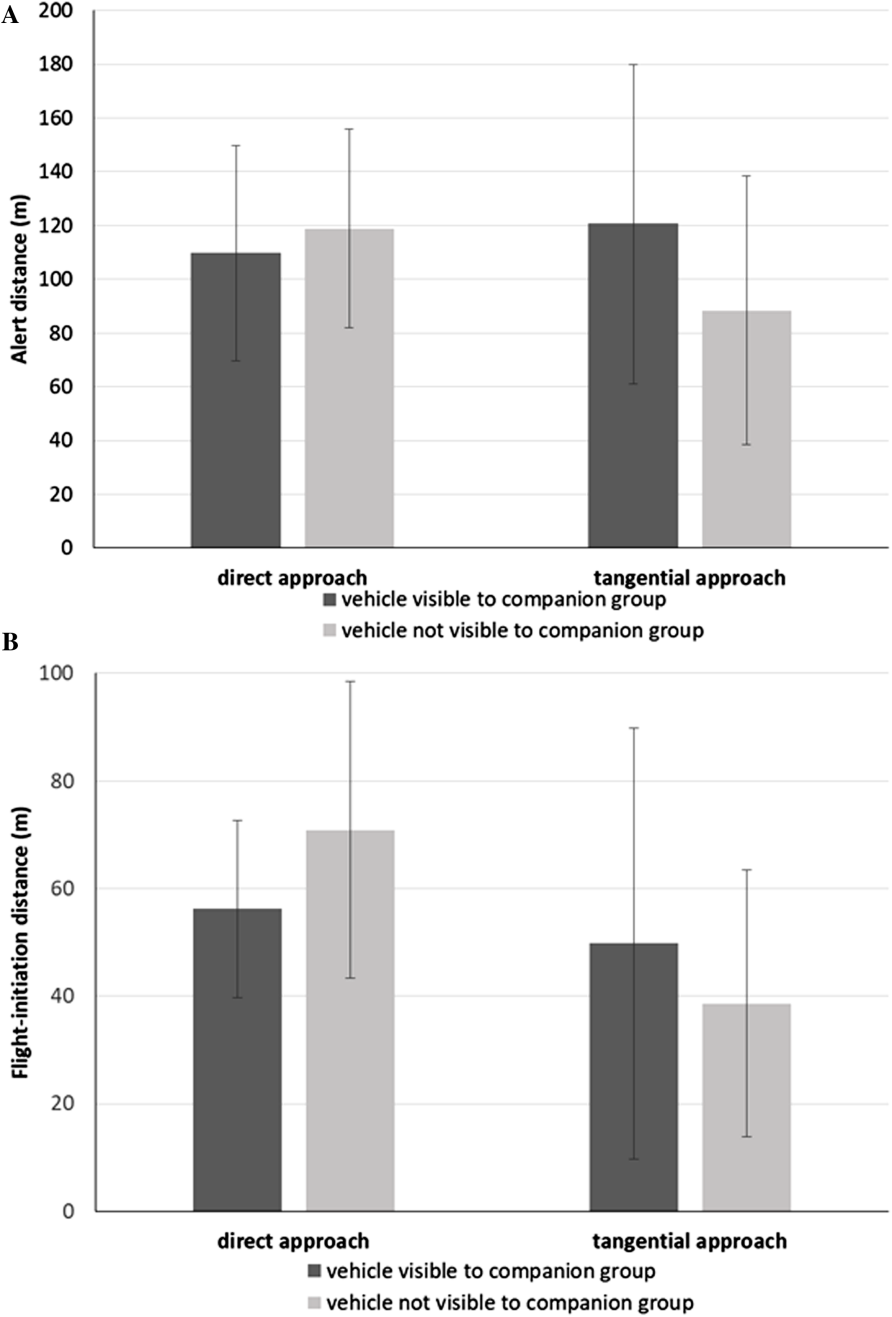
Response metrics for Canada geese exposed to vehicle approach. (A) Mean alert distance (SD) and (B) mean flight-initiation distance (SD) of a focal Canada goose relative to the direction of the vehicle approach (direct, tangential) and whether the companion group was visually exposed to the vehicle or not.

We found that the alert distance of the focal bird (Table 3) did not vary with approach direction (direct approach: $\bar{X}$ = 114.3 m, SD = 38.4 m; tangential: $\bar{X}$ = 104.5 m, SD = 56.7 m; *F*_1,10.9_ = 0.31, *P* = 0.589) or whether or not the companion group was exposed to the vehicle approach (blind: $\bar{X}$ = 103.7 m, SD = 46.1 m; exposed: $\bar{X}$ = 115.1 m, SD = 50.4 m; *F*_1,58.5_ = 0.67, *P* = 0.416), controlling for ambient light intensity (*F*_1,16.8_ = 2.34, *P* = 0.145). Instead, we found a significant interaction effect between vehicle approach direction and the degree of exposure of the companion group to the vehicle approach (*F*_1,69.1_ = 4.41, *P* = 0.040; Fig. 2A). During direct approaches, there was no significant difference in the focal bird’s alert distance whether or not the vehicle was visible to the companion group (*F*_73.6_ = 0.80, *P* = 0.426; Fig. 2A). However, during the tangential approaches, focal birds became alert at greater distances when the vehicle was visible to the companion group than when the vehicle was not (*F*_73.8_ = −2.03, *P* = 0.046; Fig. 2A). The Null Model Likelihood Ratio test indicated no effect of the random intercept for companion group ID (*χ*^2^ = 0.15, *P* = 0.929).

**Table 3:** Descriptive statistics for alert distance and flight-initiation distance (m). Descriptive statistics for alert distance (AD) and flight-initiation distance (FID) by flight-capable Canada geese exposed to vehicle approach 1–4 August 2017, at the NASA Plum Brook Facility in Erie County, OH, USA. Treatments comprised vehicle approach direction and exposure level of the companion group to the approaching vehicle.

Response variable	Approach direction	Companion group	*N*	Mean	SD	Min	Max
AD	Direct	Blind	20	118.9	37.0	57.4	191.4
	Direct	Exposed	20	109.7	40.1	46.2	181.3
	Tangential	Blind	20	88.4	50.0	0.0	165.7
	Tangential	Exposed	20	120.5	59.5	0.0	203.3
							
FID	Direct	Blind	20	70.9	27.6	29.6	146.0
	Direct	Exposed	20	56.2	16.5	31.1	87.3
	Tangential	Blind	20	38.6	24.8	0.0	87.1
	Tangential	Exposed	20	49.8	40.1	0.0	121.4

We found that the FID of the focal bird (Table 3) was affected by approach direction (direct approach: $\bar{X}$ = 63.6 m, SD = 23.6 m; tangential: $\bar{X}$ = 44.2 m, SD = 33.4 m; *F*_1,10.6_ = 6.96, *P* = 0.024). We found no effect of whether the companion group was blind or exposed to the vehicle (blind: $\bar{X}$ = 54.8 m, SD = 30.6 m; exposed: $\bar{X}$ = 53.0 m, SD = 30.4 m; *F*_1,53_ = 0.06, *P* = 0.805), after controlling for ambient light (*F*_1,15.4_ = 0.03, *P* = 0.876).

However, overriding the effect of approach direction, we found an interaction effect between approach direction and exposure of the companion group to the vehicle (*F*_1,69.9_ = 4.08, *P* = 0.047). When the vehicle was visible to the companion group, the focal bird did not vary its FID relative to the direction of the approach (*F*_32.0_ = 0.63, *P* = 0.536; Fig. 2B). However, when the vehicle was not visible to the companion group, the focal bird initiated flight at greater distances for the direct than the tangential approaches (*F*_32.0_ = 3.34, *P* = 0.002; Fig. 2B). Further, when the vehicle was not visible to the companion group, focal birds initiated flight at greater distances when approached directly, than when approached tangentially with the companion group exposed (*F*_20.6_ = 2.10, *P* = 0.048; Fig. 2B). The Null Model Likelihood Ratio test indicated no effect of the random intercept for companion group ID (df = 2, *χ*^2^ = 0.01, *P* = 0.997).

In addition, 32 focal birds (80%) exposed to direct approach scenarios opted to escape by flying ahead of the vehicle approach, whereas only four birds (10%) exposed to tangential approaches became airborne. No focal birds exposed to direct approach failed to show escape behavior, whereas 10 birds (25%) exposed to tangential approaches failed to react. In our modeling, we found a significant effect of approach direction on type of escape behavior (*F*_1,67_ = 5.83, *P* = 0.019, direct approach estimate = −3.49, SD = 1.45, tangential approach estimate = 0.00). The implication is that direct approach effected a lower probability of birds showing no escape and a higher probability of taking flight than the tangential approach. All other factors in the model were not significant: companion group exposure to the vehicle (*F*_1,67_ = 0.12, *P* = 0.732), interaction between approach direction and companion group exposure to the vehicle (*F*_1,67_ = 0.01, *P* = 0.939) and ambient light intensity (*F*_1, 67_ = 0.04, *P* = 0.851).

## Discussion

We developed an experimental scenario that controlled for multiple confounding factors (e.g., identity of animal, group size and exposure to an approaching vehicle), but allowed animals to engage in escape behavior without the constraints of an enclosure, approximating realistic animal–vehicle interactions. In this context, our main finding was that the alert and flight responses of Canada geese to vehicle approach were modulated not only by the direction of the approach, but also by whether or not the companion group could see the approaching vehicle. Interestingly, the nature of these interaction effects varied between alert distance and FID.

We found support for our first hypothesis, relative to detection of the approaching threat and perceived risk. Specifically, alert distance was not directly affected by vehicle approach direction or whether or not the vehicle was visible to the companion group. However, when vehicles approached tangentially, geese showed greater alert distances when the vehicle was visible to the companion group compared to when it was not. Further, though we found a significant effect of vehicle approach direction on FID, we also found an interaction between approach direction and the companion group. When social information was not available from the companion group, focal geese escaped at greater distances (showed greater FIDs) under direct compared to tangential approaches. However, when the companion group could see the vehicle approaching, focal birds escaped at similar distances irrespective of vehicle direction. Focal birds also escaped at greater distances under direct approach with the companion group blind to the vehicle, as opposed to tangential approaches when the companion group could see the vehicle. Such variation in the use of personal versus social information relative to the level of perceived risk has been reported in the context of predator–prey interactions (Webster & Laland, 2008; Fernández-Juricic et al., 2009), but not in animal–vehicle interactions.

Notably, our focal birds and birds composing companion groups were opportunistically taken from a larger, captive group. Thus, it is unlikely that these birds necessarily shared bonds associated with a family group (Raveling, 1969), or even the vigilance behaviors of a foraging flock (Elgar, 1989; McWilliams, Dunn & Raveling, 1994). Despite these factors, our findings lead to some interesting implications relative to animal–vehicle interactions. First, focal birds exposed to the low-risk, tangential-approach scenario adhered more so to aspects of group vigilance with regard to possible threat (Lazarus, 1978; McWilliams, Dunn & Raveling, 1994), as well as any visual signaling from the companion group (Raveling, 1969; Black & Barrow, 1985). This finding also suggests possible benefits of collective detection rather than dilution (Krause, Ruxton & Ruxton, 2002), as we controlled for the number of individuals in our experimental arena.

Second, given the position of a focal bird relative to the companion group in our design, the focal bird could be considered as on the edge of the flock, which would contribute to increased vigilance toward an immediate, oncoming risk (such as direct vehicle approach; sensu; Inglis & Lazarus, 1981), and less attention to the presence of the companion group (Creel et al., 2017). For example, animals might switch to more costly private information when there is a need to make accurate estimates, but use cheaper social information when less reliable estimates are acceptable, as per the costly information hypothesis (Boyd & Richerson, 1985; Laland, 2004). The costly information hypothesis has been supported in antipredator (Van Bergen, Coolen & Laland, 2004; Webster & Laland, 2008) and foraging contexts (Johnsson & Fredrik, 2007). Further, when perceived risk is low, prey antipredator behavior, such as vigilance and attention toward group members, can serve to generally reduce future risk (Creel et al., 2017).

In terms of escape behavior, we first found that birds exposed to direct vehicle approaches generally took flight ahead of the vehicle, whereas those exposed to tangential approaches took flight at shorter distances (i.e., either stepped to the side, ran at angles to the vehicle, or did not initiate escape). It is possible that birds exposed to direct approach not only perceived greater risk than birds approached tangentially, but also reacted as if being pursued. This conclusion is supported by multiple studies on FID with mostly humans as the approaching threat (Burger & Gochfeld, 1981, 1990; Stankowich & Blumstein, 2005; Stankowich & Coss, 2006; Møller & Tryjanowski, 2014). In other words, escape by flying ahead of the vehicle approach removed the bird from the proximity of the “predator” (Cooper & Sherbrooke, 2016), as opposed to simply sidestepping the approaching vehicle, ostensibly a more effective and energy efficient means of avoiding a vehicle on a fixed trajectory. For example, McWilliams, Dunn & Raveling (1994) observed that cackling (*Branta canadensis minima*) and Ross’s geese (*Chen rossii*) responded to eagle (primarily golden eagle, *Aquila chrysaetos*) attack by flushing into the air.

As suggested above, a vehicle approaching directly appears to conform to perceived predatory intent and the difference might indicate variation in assessment relative to risk, consistent with the threat sensitivity hypothesis (Helfman, 1989). That said, developing a vehicle-as-predator hypothesis does not account for the fact that our urban geese often failed to initiate escape in a timely manner on several occasions. We observed five incidents of focal birds being struck by a vehicle on direct approach, even at speeds of only 15.6 m·s^−1^ and nine other incidences of near misses. We suspect that these late responses to vehicles reflect the experiences of our urban geese, birds that likely experienced vehicles taking evasive actions (as they usually do in parks, etc).

For example, there is evidence that larger bird species will show greater tolerance of humans (and, presumably, human-related activities) in nonlethal situations so as to minimize energy costs from unnecessary escape and maximize resource acquisition (Samia et al., 2015). Moreover, there is evidence for bird learning with respect to risk associated with approach by automobiles (DeVault et al., 2018a). Also, birds have been shown to adjust their FID based on experience with vehicle approach, road section and posted speed, not necessarily realized or actual vehicle speed (Legagneux & Ducatez, 2013). Mukherjee, Ray-Mukherjee & Sarabia (2013) observed that American crows (*Corvus brachyrhynchos*) feeding on carrion in the same road lane as an approaching vehicle would fly or walk to the opposite lane at some point, whereas individuals in the opposite lane chose to remain in place. Finally, Nordell, Wellicome & Bayne (2017) reported that ferruginous hawks (*Buteo regalis*) that experienced vehicle approach on low-traffic volume access roads showed greater FIDs than birds exposed to vehicle approach on highways and other high-traffic volume roads, indicating possible habituation.

## Conclusions

This is the first study, to our knowledge, that examines the question of how animal alert and escape behaviors in response to vehicle approach are affected by vehicle approach direction, as well as social information. We showed that urban Canada geese interpreted risk associated with direct versus tangential vehicle approaches differently depending on the availability of social information in the flock. Our findings have two important implications.

First, there is a need for realistic experimental designs by which animal behavior in response to vehicle approach can be more realistically assessed, particularly when those behaviors affect the likelihood of animal and human mortality. We suggest that future experimental designs quantifying aspects of animal response to vehicle approach incorporate means of measuring detection/alert and flight responses, consider group effects relative to perceived risk and escape-related behaviors, quantify behavior subsequent to the initial escape response (i.e., FID) and provide opportunity for complete animal escape in response to experiment treatments (Goller et al., 2018).

Specifically, a more complete understanding of the full escape response relative to species, type of disturbance (Lima et al., 2015; Nordell, Wellicome & Bayne, 2017) and perceived risk can serve to inform management to reduce collision frequency under varying vehicle-traffic scenarios. For instance, an animal that simply steps to the side of an approaching vehicle or one that escapes, but remains within a hazardous area (traffic margin or airfield) still poses a collision risk. Escape behaviors can be exploited to enhance responses (e.g., Blackwell & Seamans, 2009; Doppler et al., 2015) and a priori escape distances required for surviving a vehicle approach (based on species behavior and vehicle approach speeds) can inform planning, such as location of designated cover or safe areas.

Second, escape responses can be greatly affected by the availability of social information about the vehicle approach. Our findings suggest that when that social information is available, the effects of heightened risk associated with a direct approach might be reduced, leading to the animal delaying the escape, which could ultimately increase the chances of a collision. However, we note that in our design our companion group was confined to an enclosure. It is possible that had these birds been allowed to escape in the same way as the focal bird, the response might have been different.

Future studies should assess how information about a vehicle, rather than a predator/human observer, flows within a flock. Such studies should include aspects of vehicle speed and size, which affect the rate of image expansion (i.e., that of the approaching vehicle) on the retina of the animal, or looming (DeVault et al., 2015; Blackwell et al., 2019). In addition, because an animal’s behavioral and physiological profiles will change with season, we suggest that a seasonal component would be interesting to assess relative to alert distance and FID. A better understanding of animal response to vehicle approach relative to realistic scenarios and information exchange within groups will provide important insights to predicting animal responses to vehicles.

## Supplemental Information

10.7717/peerj.8164/supp-1 Supplemental Information 1 Raw data.

10.7717/peerj.8164/supp-2 Supplemental Information 2 SAS code.

## Additional Information and Declarations

### Data Availability

The following information was supplied regarding data availability: The raw data and code are available in Supplemental Files.
