# Supplementary material for: Social information affects Canada goose alert and escape responses to vehicle approach: implications for animal–vehicle collisions
Source: PeerJ. 2019 Dec 17;7:e8164. doi: 10.7717/peerj.8164 (PMC6924344; doi:10.7717/peerj.8164)
Supplement: Supplemental Information 2 [file peerj-07-8164-s002.docx]

**Code S2**. SAS code used for the statistical analyses.

Flushing of focal birds

data cago17;

input date $ run $ direction $ cgtreat $ flushed $ cgID ad fid;

‘comment run = replicate, cg = companion group, cgtreat = blind or exposed, cgID = identity of the companion group, ad = alert distance, fid = flight-initiation distance’;

;

cards;

;

proc sort data = cago17;

by flushed direction cgtreat ad fid;

run;

;

proc means data = cago17;

by flushed direction;

var ad fid;

run;

;

proc means data = cago17;

by flushed cgtreat;

var ad fid;

run;

;

proc means data = cago17;

by flushed;

var ad fid;

;

proc npar1way data = cago17;

class flushed;

var ad;

run;

;

proc npar1way data = cago17;

class flushed;

var fid;

run;

;

proc mixed data = cago17;

class flushed direction cgtreat cgID;

model ad = flushed direction cgtreat direction*cgtreat/ ddfm = kr chisq solution outp = resids;

random int / subject = cgID type = ar(1);

run;

;

proc plot data=resids;

plot resid*pred;

run;

proc univariate data = resids plot normal;

var resid;

run;

;

proc mixed data = cago17;

class flushed direction cgtreat cgID;

model fid = flushed direction cgtreat direction*cgtreat/ ddfm = kr chisq solution outp = resids;

random int / subject = cgID type = ar(1);

run;

;

proc plot data=resids;

plot resid*pred;

run;

;

proc univariate data = resids plot normal;

var resid;

run;

Run time (Time for the driver to cover the 251 m)-See definitions, above.

data cago17;

input date $ run oldcode $ direction $ cgtreat $ runtime runtimeadj light cgid;

comment runtime is uncorrected for evasive actions by driver;

cards;;

proc sort data = cago17;

by direction;

run;

;

proc means data = cago17;

by direction;

var runtime runtimeadj;

run;

;

proc sort data = cago17;

by cgtreat;

run;

;

proc means data = cago17;

by cgtreat;

var runtime runtimeadj;

run;

;

proc sort data = cago17;

by direction cgtreat light cgid;

run;

;

proc mixed data = cago17;

class direction cgtreat light cgid;

model runtime = direction cgtreat direction*cgtreat / solution cl outp = resids ddfm = kr;

random int / subject = cgid type = ar(1);

lsmeans direction cgtreat direction * cgtreat/pdiff;

run;

;

proc plot data=resids;

plot resid*pred;

run;

;

proc univariate data = resids plot normal;

var resid;

run;

;

proc mixed data = cago17;

class direction cgtreat light cgid;

model runtimeadj = direction cgtreat direction*cgtreat / solution outp = resids ddfm = kr;

random int / subject = cgid type = ar(1);

lsmeans direction cgtreat direction*cgtreat/pdiff;

run;

;

proc plot data=resids;

plot resid*pred;

run;

;

proc univariate data = resids plot normal;

var resid;

run;

;

Main analyses-see definitions, above.

data cago17;

input date $ run direction $ cgtreat $ escape wind temp light ad fid cgid;

;

cards;

;

proc sort data = cago17;

by direction cgtreat cgid wind temp light;

run;

;

proc means data = cago17;

class direction cgtreat;

var wind temp light;

run;

;

proc univariate normal plot data = cago17;

var wind temp light;

run;

;

proc npar1way data = cago17;

var wind temp light;

class direction;

run;

;

proc npar1way data = cago17;

var wind temp light;

class cgtreat;

run;

;

proc sort data = cago17;

by direction cgtreat cgid wind temp light;

run;

;

proc means data = cago17;

class direction cgtreat;

var ad fid;

run;

;

proc means data = cago17;

class direction;

var ad fid;

run;

;

proc means data = cago17;

class cgtreat;

var ad fid;

run;

;

;

proc mixed data = cago17;

class direction cgtreat cgid;

model ad = direction cgtreat direction*cgtreat light / solution cl outp = resids ddfm = kr;

random int / subject = cgid type = ar(1);

lsmeans direction cgtreat direction * cgtreat/pdiff;

run;

;

proc univariate normal plot data = resids;

var resid;

run;

;

;

proc mixed data = cago17;

class direction cgtreat cgid;

model fid = direction cgtreat direction*cgtreat light / solution cl outp = resids ddfm = kr;

random int / subject = cgid type = ar(1);

lsmeans direction cgtreat direction * cgtreat/pdiff;

run;

;

proc univariate normal plot data = resids;

var resid;

run;

;

;

proc glimmix data=cago17 empirical=mbn method=quad;

class direction cgtreat cgID;

model dependent = direction cgtreat direction*cgtreat light /dist=mult link=clogit solution or(label);

random int / subject=cgid;

store gmxres;

run;
